# Comparative dataset of experimental and computational attributes of UV/vis absorption spectra

**DOI:** 10.1038/s41597-019-0306-0

**Published:** 2019-12-05

**Authors:** Edward J. Beard, Ganesh Sivaraman, Álvaro Vázquez-Mayagoitia, Venkatram Vishwanath, Jacqueline M. Cole

**Affiliations:** 1 Cavendish Laboratory, Department of Physics, University of Cambridge, J. J. Thomson Avenue, Cambridge, CB3 0HE UK; 2 ISIS Neutron and Muon Source, STFC Rutherford Appleton Laboratory, Harwell Science and Innovation Campus, Didcot, Oxfordshire OX11 0QX UK; 3 Argonne National Laboratory, 9700 South Cass Avenue, Lemont, IL 60439 USA; 4 Department of Chemical Engineering and Biotechnology, University of Cambridge, West Cambridge Site, Philippa Fawcett Drive, Cambridge, CB3 0FS UK

**Keywords:** Cheminformatics, Materials for optics, Optical materials, Computational methods

## Abstract

The ability to auto-generate databases of optical properties holds great prospects in data-driven materials discovery for optoelectronic applications. We present a cognate set of experimental and computational data that describes key features of optical absorption spectra. This includes an auto-generated database of 18,309 records of experimentally determined UV/vis absorption maxima, *λ*_*max*_, and associated extinction coefficients, *ϵ*, where present. This database was produced using the text-mining toolkit, ChemDataExtractor, on 402,034 scientific documents. High-throughput electronic-structure calculations using fast (simplified Tamm-Dancoff approach) and traditional (time-dependent) density functional theory were executed to predict *λ*_*max*_ and oscillation strengths, *f* (related to ϵ) for a subset of validated compounds. Paired quantities of these computational and experimental data show strong correlations in *λ*_*max*_, *f* and *ϵ*, laying the path for reliable *in silico* calculations of additional optical properties. The total dataset of 8,488 unique compounds and a subset of 5,380 compounds with experimental and computational data, are available in MongoDB, CSV and JSON formats. These can be queried using Python, R, Java, and MATLAB, for data-driven optoelectronic materials discovery.

## Abstract (summary)

**Table:** 

Measurement(s)	ultraviolet–visible spectrum • absorption wavelength • extinction coefficient
Technology Type(s)	digital curation

Machine-accessible metadata file describing the reported data: 10.6084/m9.figshare.10304897

## Background & Summary

Progress in materials science is driven by the publication of articles in scientific journals where results are presented in tables, figures and continuous prose. The ever-growing size of this corpus and extensive back catalog of papers has made it difficult for scientists to inform their research using all the available data. Due to advances in natural language processing (NLP) and machine learning (ML) techniques, the core textual information from these articles can now be extracted automatically from papers at speeds much greater than can be achieved manually. However, the success of NLP-based text-mining tools is predicated on the extent by which the tool is tailored to the field of research in which documents are mined, since each research domain uses highly specialist language and labeling that confounds generic NLP-based text-mining tools. Fortunately, NLP-based tools, such as ChemDataExtractor^1^, have been designed for auto-extracting data from the materials science domain, and have already been used to auto-generate databases comprising experimental data of chemical compounds and their Curie and Néel magnetic phase-transition temperatures^2^. Kim *et al*. have also demonstrated how to auto-extract materials databases on synthesis parameters^3^.

Material databases containing results from *ab initio* computational calculations have also been assembled in the fields of organic photovoltaics^4^ and batteries^5^. Such databases stand to be highly complementary to those comprising experimental data, particularly when considered in the context where paired quantities of cognate experimental and computational data could be combined. A databank of experimental data could be used to benchmark high-throughput *ab initio* quantum-chemical calculations on cognate computational data. By comparing each computational result to an experimental reference, an internally consistent reliability measure of the calculation is afforded. Pending a good match between experiment and computation, the associated wavefunction can be considered to be reliable. At that point, the computational approach can be used to calculate properties that were not reported by experiment. Meanwhile, the experimental data are naturally restricted to the measurement information that is available in the original paper from which they were extracted. Thus, the availability of materials databases that comprise cognate experimental and computational data would place computational calculations in an advantageous position, whereby their associated wavefunctions could be used to proliferate many more data, with the confidence that these data would be reliable; as such, the database would be further enriched with appropriate information.

**Fig. 1:**
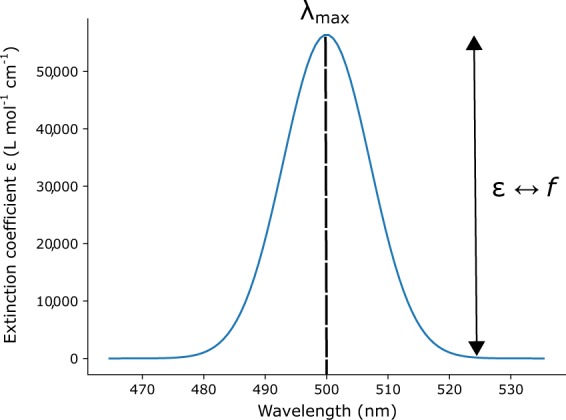
A simple UV/vis absorption spectrum displaying the peak absorption wavelength, *λ*_*max*_, whose intensity is given by the molar extinction coefficient, *ϵ*, whose computational analog is the oscillation strength, *f*.

Given this potential vantage point for computational data, the forging of a pipeline that auto-generates materials databases of cognate experimental and computational data was deemed to be strategically useful. Realizing this goal is the subject of this paper, whereby we present a new materials database of UV/vis absorption spectral attributes^6^ whose experimental data component has been auto-generated by mining text from documents in the scientific literature and pertains to: a chemical material, its peak absorption wavelength(s), *λ*_*max*_, and the molar extinction coefficient of each peak, *ϵ*. These data are coupled to the results of a computational pipeline that uses fast (approximating) and slow (traditional) quantum-chemical methods, within a high-throughput computational framework, to produce the comparable UV/vis absorption spectral metrics, *λ*_*max*_, and the oscillation strength, *f*, a metric related to *ϵ* (see Fig. 1). Such data were selected because there is no large database publicly available on these property attributes even though UV/vis absorption spectroscopy is such a fundamental materials characterization tool. The largest commercial offering comes from Reaxys (https://www.reaxys.com), a manually curated database containing 886,213 compounds with a max spectra peak greater than 0. However, there are only a few open source manually curated databases of UV/vis absorption spectral parameters available, that are all very modest in size. For example, the NIST Chemistry Webbook^7^, the DSSCDatabase^8^ and the Max Weaver dye library^9^ contain UV/vis absorption data on 600, 4,400 and 2,700 compounds, respectively. As the scientific literature holds such a vast knowledge base about chemicals and their UV/vis absorption spectral properties, the auto-generated nature of our materials database^6^ is scalable and larger than these manually curated databases^7–9^ and it is not susceptible to human error^10^. Our data repository^6^ offers good reuse value, as it will enable the option for ML techniques to be applied to the database, to identify patterns in the data that represent underlying relationships between chemical structure and UV/vis absorption spectral properties. Such structure-property relationships can be employed to shortlist promising material candidates for bespoke applications. To this end, a sub-set of this database has already been used to discover photovoltaic chromophores^11^. This trend moves towards the ultimate goal of data-driven materials discovery for optical and optoelectronic applications.

## Methods

**Fig. 2:**
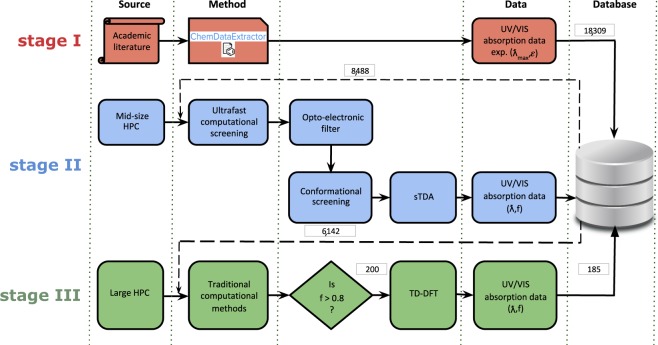
The workflow associated with different stages of data processing. Stage I (top row): ChemDataExtractor extracts chemical information from the academic journal. Stage II (middle row): Unique chemical entries from the MongoDB server are passed through a fast-screening layer. Stage III (bottom row): Best candidates are identified and TD-DFT calculations are performed for those select cases. All of the stages utilize a secure MongoDB server for database management. Numbers enclosed in rectangular boxes indicate the number of data samples entering or leaving a stage.

This section presents a summary of the data acquisition and processing methods. An overview of the operational workflow that generates the materials database of UV/vis absorption spectral properties^6^ is given in Fig. 2.

### Stage I: acquisition of experimental database using ChemDataExtractor

#### Acquiring a corpus

The experimental data acquisition process (Stage I) is described visually on the first row of Fig. 2. A corpus of 387,878 articles from 33 different journals was generated using a series of purpose-built web-scraping tools. The tools were designed to download all articles from the web pages of the Royal Society of Chemistry and Elsevier using a list of relevant journals known to contain organic compounds (a full journal list is provided in the Supplementary Information). An additional 14,156 articles were obtained from the Springer website using the ‘scrape’ package from ChemDataExtractor, version 1.3, through the use of the case-insensitive search query ‘uv + vis’. These publishers were chosen for their text and data mining policies, which allow the large scale extraction of data for non-commercial purposes. Only HTML and XML article formats were included in the data extraction by restricting the download to articles released after the year 2000. For all cases, the tools were designed to satisfy the journal-specific Text and Data Mining (TDM) terms and conditions of each publisher, and make use of their Application Programming Interface (API) where appropriate. All downloaded articles contained the complete text and were saved in HTML format. Each article was also tagged with its unique Digital Object Identifier (DOI), enabling any inadvertent duplicates of a given article to be avoided.

#### Data extraction

The chemical records were extracted from the complete corpus of 402,034 articles using ChemDataExtractor, version 1.3, which was used in its default configuration except where stated otherwise, in that it was altered to deal with certain parts of this process. ChemDataExtractor converts HTML articles into a standard structure for rule-based phrase parsing and extracts the core chemical information^1^. Given the large quantity of data sought from many articles, this task was achieved by porting ChemDataExtractor into a workflow optimized for parallelized data extraction, so that it could be executed using the petaflops-class high performance computing (HPC) resource, Theta, at the Argonne Leadership Computing Facility (ALCF). The experimental data were initially extracted using the UV/vis phrase-parser package in ChemDataExtractor, yielding 18,309 individual instances of {compound, *λ*_*max*_} paired data. For an evaluation subset of 19 journals, only 26.4% of the max values initially extracted were accompanied by a recording of their molar extinction coefficient, *ϵ*, the units for which were available from only 3.2% of the total dataset. The origin of the significant under-performance of these two metrics was found to be due to the table processor of ChemDataExtractor, which cannot parse certain tabulated representations of cognate {*λ*_*max*_, *ϵ*} pairs.

The logic in ChemDataExtractor version 1.3 scans each heading cell for specific keywords for categorization. Once it has found these keywords, it triggers the extraction of the core data from the table rows, cell by cell. The original UV/vis parsers in ChemDataExtractor were built to identify three different types of UV/vis column titles; where they contained *λ*_*max*_, *ϵ*, or both together. For cells containing just *λ*_*max*_ or *ϵ* data, the table extraction logic would then parse each individual row in succession, assigning the values on each row to a common chemical record and pairing the *λ*_*max*_ and *ϵ* data as a single ‘peak’ object. This pairing worked well for combining data in cases where each cell contained a single *λ*_*max*_ or *ϵ* but failed when multiple values for either data specifier were housed inside a single cell eg. a cell whose data entry is {${\lambda }_{max}^{1}$, ${\lambda }_{max}^{2}$, ${\lambda }_{max}^{3}$} or {*ϵ*^1^, *ϵ*^2^, *ϵ*^3^}. For these cases, the default behavior of the algorithm stored these as an ordered list of separate isolated ‘peak’ objects. Post-processing logic was consequently added to identify these cases using a number of metrics, including the query: is the number of ‘peak’ objects containing solely *λ*_*max*_ data equal to that containing solely *ϵ* data. If the result of this query was found to be true, the data were paired up according to their index, *i*, in the list i.e. {${\lambda }_{max}^{i}$, *i*}.

Additional rules were added to the table header parsers to allow more variation in the units of the molar extinction coefficient and to include standard form and logarithmic units, indicated by the presence of ‘×10^*n*^’ or ‘log’, respectively. Having incorporated these changes into an altered form of ChemDataExtractor, it was re-tested on the evaluation subset. The number of UV/vis peak objects that this test identified, which contain cognate pairs of *λ*_*max*_ and *ϵ* (where all extinction coefficients had units) and an associated compound, was found to have increased from 782 to 4,181 (i.e. augmented by a factor of 5.3), relative to the associated performance metrics of the initial evaluation using results from ChemDataExtractor version 1.3.

#### Post-processing and storage

Following data extraction, a subroutine was run to standardize all chemical names. This subroutine used the National Cancer Institute’s Chemical Identifier Resolver (CIR) through their python wrapper, CIRpy (https://github.com/mcs07/CIRpy), to convert the chemical names into the simplified molecular-input line-entry system (SMILES) notation^12^; this in turn used the NLP tool, OPSIN^13^, in conjunction with a database lookup. The Cooley computer cluster at the ALCF was employed for these chemical-name resolution tasks. The extracted data were then hosted as a NoSQL database within a MongoDB data management framework which was chosen for its flexible data format with JSON-like ‘document’ objects, and the variety of its allowed query parameters.

Queries were sent to this database to select all compounds resolved into SMILES format containing a ‘peak’ object with at least one *λ*_*max*_. Where available, *ϵ* and all associated units were also extracted alongside the solvent information of the compound. The chemical set of these experimental data were used as a starting point for quantum-chemical calculations that were performed on the same compounds.

### Stage II: data filtering and fast computational screening

#### Data filtering

The experimental data from stage I were fed into stage II which is represented by the middle rows of the operational workflow shown in blue within Fig. 2. A total of 8,488 unique chemical compounds were isolated from the original experimental output based on their international chemical identifier code (InChiKey). A raw SMILES string obtained from stage I is preprocessed, and canonicalized (step 1 below). An InChiKey was generated from the canonicalized smiles using RDKIT (https://www.rdkit.org). InChiKey labels support tracking of multiple instances of recorded compounds; for example, compound ‘X’ with UV/vis absorption spectral properties could be reported multiple times across the scientific literature, in which case ChemDataExtractor would store them as separate instances. All instances of a compound with a valid unique InChiKey were recorded alongside a DOI referencing its original scientific publication. These data were then passed through a rule-based opto-electronic filter to perform the following operations (in order):

1. Remove invalid character strings from incoming chemical structures represented within a SMILES notation and canonicalize them using Open Babel^14^. For example, the string ‘[<S>]’ is considerd invalid and is removed due to the presence of the < and > characters, which cannot be parsed by the SMILES resolution software.
2. Filter out compounds containing heavy metals or charged species. This restricts high-throughput calculations of electronic structures to a realistic goal of producing reliable wavefunctions for organic compounds.
3. Identify significant regions of *π*-conjugation by looking for aromatic cycles, double bonds or a combination of both in the canonicalized SMILES string.
4. Where relevant, trim long alkyl chains to methyl groups which should have negligible effects on optical absorption^15^. This minimizes computational cost while not compromising scientific results.
5. Avoid computing molecules that were too complex or too small to run using the high-performance computing resource. This decision was made by considering the total number of electrons (NEL) in the molecule. Molecules were divided into four different categories; small (50 ≤ NEL ≤ 140), medium (141 ≤ NEL ≤ 220), large (221 ≤ NEL ≤ 300) and extra large (301 ≤ NEL ≤ 370). Electronic structure calculations for compounds with (NEL > 370) were deemed too expensive to compute in a manner that would lead to convergence within the allocated computing time. Even if a particular compound was not computed, all the relevant chemical information and extracted experimental records were still made available in the dataset. It is noted that the removal of complex dyes where NEL > 370 may result in a slight bias towards redder compounds.

This filtering process reduced the input set of 8,488 compounds to a set of 6,142 compounds, which were each then assigned a HPC band based on its NEL value. A ‘FILTERED’ tag was added to each compound that satisfied all five requirements and all data generated for this subset were added to a separate branch of the JSON document tree.

These compounds were then passed through a pipeline incorporating a Quantum Mechanical WorkFlow (QMWF), which can perform ensemble jobs that employ a wide range of diverse quantum-chemical methods and computational software platforms, across versatile HPC installations (source code: https://github.com/alvarovm/qmwf). As a first step, 3D structures were generated from SMILES strings using the RDKit software package^16^. From a random pool of 1,500 conformations, five of the most stable and geometrically diverse structures, energies and forces were evaluated with the force field MMFF94 in RDKIT. The results were parsed and attributed a ‘conformers’ tag within the current branch of the JSON document. QMWF then invokes the MOPAC (Molecular Orbital PACkage, http://openmopac.net) semi-empirical computational software to perform PM7 semi-empirical calculations^17^ (each data record contains the MOPAC version used under the ‘version’ key). These calculations were used to screen low-energy molecular conformations for each chemical compound. Ground-state calculations were performed on the lowest-energy conformer and given a ‘mopac’ tag within this branch of the JSON document. Each lowest-energy conformer generated by MOPAC was then exported to the ORCA software platform^18^ wherein its molecular geometry was optimized using density functional theory (DFT)^19,20^, through a double zeta basis set and a PBEh-3c exchange-correlation method^21^. The simplified Tamm-Dancoff density functional theory approach (sTDA)^22^ was then applied to the geometry-optimized structure, which is ideal for accessing excited-state properties of molecular systems that possess large numbers of atoms (~500–1,000). A hybrid wB97X-D3 exchange-correlation method along with triple zeta basis sets were employed for this step. These calculations afforded *λ*_*max*_ and *f* properties for each molecule which were parsed and added to the ‘orca’ tag within this branch of the JSON document.

### Stage III: applying traditional computational methods to chemicals with the most promising UV/vis absorption spectral properties

The results generated by sTDA were analyzed to identify compounds with strong UV/vis absorption characteristics. The first excitation of a molecule corresponds to its largest wavelength of absorption, as wavelength is inversely proportional to excitation energy as a consequence of the Planck–Einstein relation. Accordingly, the database of sTDA results was queried to select compounds whose first excitation possess a large oscillator strength, as shown in the bottom row of Fig. 2. A total of 1,302 compounds were found to exhibit a first excitation with an oscillator strength *f* > 0.8. 200 of these compounds were randomly selected and subjected to full time-dependent density functional theory (TD-DFT) calculations, using the NWChem package^23^, in order to validate the sTDA results. Geometry optimizations used the B3LYP functional and 6–31 + G* basis sets while TD-DFT computations employed the LRC-wPBEh functional and 6–311 + G* data sets. 185 out of the 200 compounds were found to converge, from which a number of properties were retrieved at each stage of the workflow; such properties included geometries, total energies, dipole moments, oscillator strengths, transition dipole moments, and orbital energies. The corresponding data were subsequently added to the JSON tree with a key and some tags, where the key describes the stage of the calculation that generated the data and the tags are sub-dictionaries of arrays of the parsed information (e.g. all of the TD-DFT parsed information were added under an ‘nwchem’ tag).

An example ‘uvvis’ tag with only one entry.

## Data Records

A static version of the described database can be downloaded from figshare^6^. The overall format of the data records is described in Table 1. Each data object contains several fields about the experimental and computed properties of the compounds. The ‘inchikey’ provides a unique identifier for each compound which can be used to filter out duplicate data from the database^6^. A ‘PRISTINE’ tag holds all of the original data records parsed from ChemDataExtractor, including the SMILES strings (‘SMI’ tag) and the experimental UV/vis absorption spectral property values (‘uvvis’ tag). Each unique set of experimental values (‘peaks’ sub-tag) are assigned as values to the ‘uvvis’ tag, along with a ‘doi’ key that refers to the DOI of the scientific paper from which the set of data was extracted. An example is shown above for the datum with ‘inchikey’: *‘WAJKAWOYYMLWNI-UHFFFAOYSA-N’*.

**Table 1:** Description of data records.

Key	Description	Data type
inchikey	International chemical identifier key	String
doi	Source document DOI	String
lambda	Experimental value of wavelength	Float
lambda_unit	Reported unit of wavelength	String
extinction	Extinction coefficient	Float
extinction_unit	Reported unit of extinction coefficient	String
solvent	Solvent reported in the source document	String
amplitude	Computed value of wavelength	Float
oscillator_strength	Computed value of oscillator strength	Float

As described in Stage II, a ‘FILTERED’ tag is added to those compounds which are accepted by the HPC filtering stage. Compounds processed through this stage are very rich in information from different levels of theory and each sub-stage is assigned with a value by the ‘FILTERED’ tag. The compounds that were selected for sTDA excited-state calculations carry an ‘orca’ tag. Similarly, a compound that reached the final stage of screening carries a ‘nwchem’ tag. Within each stage, an ‘excited_states’ tag keeps a detailed record of the excited states, by means of an ‘orbital_energy_list’ sub-tag. An example for a single-orbital energy list is shown below.

An example ‘orbital_energy_list’ tag value.

The original data reported in the publication are retained without modification. For example, if cm^−1^ is the original unit reported for the ‘lambda_unit’ tag, then the database record^6^ would reflect this. The only exception is when an empty field is encountered for experimental values that have been parsed by ChemDataExtractor, for which a ‘NULL’ value is assigned to the associated keys. For the purpose of tracking the information back to source, every ‘peaks’ tag found inside the ‘uvvis’ tag has an associated ‘doi’ key. The data records^6^ are made available in MongoDB, JSON and CSV format, although there is far more information in the MongoDB and JSON formats, than in the CSV format, owing to the complex nested structure of the dataset.

## Technical Validation

**Fig. 3:**
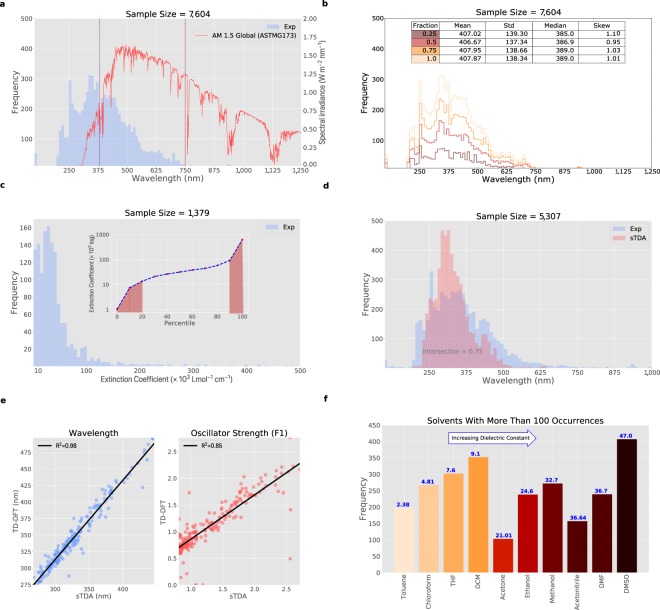
Data validation. (**a**) Histogram of experimental *λ*_*max*_ values for all valid compounds in the dataset^6^ (blue) overlaid with the AM 1.5 Global Tilt Spectra (red). (**b**) Histogram for different fractions drawn from the experimental *λ*_*max*_ values for all valid compounds in the dataset^6^. (**c**) Histogram for experimental extinction coefficients, *ϵ*, for all valid compounds in the dataset^6^ (inset: experimental extinction coefficient percentiles with the outliers outlined in red). (**d**) Histograms for a subset of compounds of their experimental *λ*_*max*_ values (blue) and computed first excitation wavelengths ${\lambda }_{max}^{1st}$ (red). (**e**) Comparison of sTDA computed properties with the corresponding values computed by TD-DFT. (**f**) Bar chart of the 10 most common solvents used in the experimental measurements of *λ*_*max*_ and *ϵ* values, where there are at least 100 occurrences in the database^6^; solvents are ordered according to the increasing values of dielectric constants. NB: Units in plots correspond to those found most frequently during our data extraction.

A major goal of this study is to provide a reliable, high-quality dataset of UV/vis absorption spectral properties of chemicals for the scientific community. To discuss the accuracy of our dataset^6^, the most relevant attributes and validation metrics have been described in Fig. 3. Out of 8,488 unique chemical compounds isolated from the original experimental dataset^6^, 7,726 compounds were found to have valid experimental values with at least one *λ*_*max*_ recorded from a UV/vis absorption spectrum. The remaining 762 were false positives of {chemical, *λ*_*max*_} pairs which had been incorrectly assigned using ChemDataExtractor version 1.3. These were omitted once the UV/vis targeted version of ChemDataExtractor was implemented (described in detail in Stage I); while this reduced the total number of results by 8.6%, it naturally improved the overall precision of the data-extraction process. However, the SMILES forms of the original 8,488 compounds were parsed through the computation screening stages (II-III) for the purpose of completeness, and consequently these 762 compounds contain computationally-derived *λ*_*max*_ values but no experimental counterpart.

7,604 of the 7,726 compounds have *λ*_*max*_ values of less than 1,200 nm, with a distribution shown in Fig. 3a by the blue histogram which is split into bins with width of 12 nm. 7,361 of these 7,604 compounds shown absorb UV/vis light, 190–750 nm; the dashed lines of Fig. 3a partition this light into UV (190–380 nm; left of purple dashed line) and visible (380–750 nm; between dashed lines) regions. Overlaid in red is the AM 1.5 Global Tilt Spectra^24^ which represents the light emission profile of solar radiation, incident on the Earth’s surface at a slope of 37° to account for atmospheric scattering and absorption. Compounds whose *λ*_*max*_ values are coincident with a wavelength at which sunlight emits (310–750 nm) have potential use as sunlight harvesters for applications such as photovoltaics.

Data in the entire region presented in Fig. 3a are also relevant to the wider field of optoelectronics and color chemistry. The color distribution of these data is skewed such that a greater density of compounds absorb at the lower wavelengths, particularly where *λ*_*max*_ < 550 nm. This skew indicates that our database^6^ provides a representative set of organic chromophores, since the majority of organic colorants appear red, yellow or orange (i.e. absorbing green-violet light), while colorants that appear violet (*λ*_*max*_ ~ 550–600 nm) or blue (*λ*_*max*_ ~ 600–700 nm) are naturally very rare^25,26^. Nonetheless, there are still 550 compounds in our dataset^6^ whose *λ*_*max*_ values lie in the 600–750 nm region of light and thus manifest as blue chromophores. It is worth noting that these instances of blue colorants in our database^6^ contrast starkly with those from the Max Weaver dye library where blue represents the highest number of textile dyes for a single color^9^.

A check was also made that our dataset of 7,604 compounds^6^ was of a sufficient size to present a representative distribution of *λ*_*max*_ values. To this end, core statistics of the distribution shown in Fig. 3a were compared against those from three randomly sampled data subsets that contain $\frac{1}{4}$, $\frac{1}{2}$ and $\frac{3}{4}$ of the total dataset. Histograms of the resulting distributions are shown in Fig. 3b, overlaid against the total dataset. Visual inspection of these results shows clearly that the essential features of each histogram are preserved. Figure 3b (Table inset) also displays core statistics of each distribution: mean, standard deviation (std), median, and coefficient of skewness (skew)^27^. These were calculated to serve as quantitative evaluation metrics for this comparison. The mean and median *λ*_*max*_ values calculated for the different histograms span ranges of 3.58 nm and 6 nm, respectively; this range is very modest, being about two orders of magnitude (0.9% and 1.5%) of the average values themselves. The absence of significant relative variation in these quantities indicates that the dataset is sufficiently large to represent the average distribution of UV/vis absorption peak wavelength data for organic compounds in the scientific literature. A similar argument can be made that the spread of data is representative of the distribution for organic compounds in the literature, via analysis of the standard deviation and skew metrics; their respective spans (7.59 and 0.05) correspond to 5–6% and 5% of their absolute values. All distributions in Fig. 3b naturally exhibit a positive skew owing to the long tail of the distribution at higher *λ*_*max*_ values, where there are fewer compounds that absorb in this range.

Figure 3c displays the distribution of 1,379 molar extinction coefficients that were extracted from the scientific literature, wheresoever they presented together with cognate *λ*_*max*_ values that belong to the dataset of 7,604 organic compounds^6^, and where their values lie within the range 1 × 10^3^–5 × 10^5^ Lmol^−1^ cm^−1^; values outside of this range were considered to be in error owing to their unrealistic values and so were omitted as statistical outliers. The *ϵ* values in this histogram are presented using a bin size of 6.25 × 10^3^ Lmol^−1^ cm^−1^. The accompanying plot (inset) shows the logarithm of *ϵ* as a function of increasing magnitude of *ϵ* which is given in the form of a rank order, i.e. the 0 and 100% percentile represent the smallest and largest values of *ϵ,* respectively. This plot reveals that the majority (20–90% percentiles) of data lie within the range 10^4^–10^5^ Lmol^−1^ cm^−1^, where the values track a linear trend with rank order as one would expect for a representative distribution of *ϵ* values: the *i*^*th*^ rank ordered *ϵ* value should increment in small, continuous, linear steps across the general population of organic compounds. The observed sudden and substantial (logarithmic) nature of the deviation from linearity at both percentile extremes (<20%; >90%) suggests the presence of a data irregularity. This irregularity was diagnosed as being due to missing or incorrect assignments of the exponent used in the standard form that is typically used to represent *ϵ*. These irregularities would explain the long, but low-frequency, tail of outliers observed beyond 10^5^ Lmol^−1^ cm^−1^ in the histogram of Fig. 3c, as well as the bimodal appearance of this histogram, whereby the lowest 20% percentile accounts precisely for the sum of the frequencies (135 + 141) for the two bins that afford the modal distribution where *ϵ* < 10^5^ Lmol^−1^ cm^−1^ (*cf*. 276/1379 × 100 = 20.01%). Thus, a truly representative distribution of *ϵ* values is likely to be unimodal with a positive skew. Yet, values from all percentiles shown are retained in the dataset^6^ to safeguard the most poignant information about *ϵ* since its error appears to lie purely within a mislabeled exponent while the value is otherwise correct, and an incorrect exponent can be re-estimated quite readily using simple logic, e.g. by identifying an incorrect exponent from its percentile value, and adopting a new exponent from that of a similar compound which is known to be correct. All extinction coefficient percentiles lower than the 20^*th*^ percentile and greater than the 90^*th*^ percentile have been flagged as red in the subplot of Fig. 3c, to indicate that caution is required when considering the value of their exponents.

Having verified these experimental data on *λ*_*max*_ and *ϵ* values, extracted from UV/vis absorption spectra of organic compounds, a comparison with cognate computationally-derived data was performed. Figure 3d compares the distribution of experimental *λ*_*max*_ values for 5,307 compounds with that of their cognate first excitation wavelengths, ${\lambda }_{max}^{1st}$, computed using sTDA. The sTDA distribution of ${\lambda }_{max}^{1st}$ exhibits a systematic bias towards lower wavelengths (i.e. higher energies), relative to the distribution of experimental *λ*_*max*_ values. This stands to reason since the first-excitation nature of these sTDA calculations results in UV/vis absorption peak values, ${\lambda }_{max}^{1st}$, exhibiting the lowest possible wavelength; while higher-order excitation wavelengths will naturally afford lower wavelengths.

As previously described, each compound in our dataset^6^ must possess at least one valid experimental *λ*_*max*_ value, but it may in fact contain multiple UV/vis absorption peaks. The intersection area of the two overlapping histograms was computed, as shown in Fig. 3d. Histogram intersection measures the similarity between two histograms, with a value between 0 (i.e. no overlap, no similarity) and 1 (i.e. identical). A systematic bias of ${\lambda }_{max}^{1st}$ towards lower wavelengths, relative to the *λ*_*max*_ experimental distribution is observed. This is indicative of the nature of the sTDA calculation since it outputs the lowest possible excitation wavelength. A 75% overlay between computational and experimental results is nonetheless reassuring, especially when considered in light of the fact that the calculations are all gas-phase models; secondary factors such as solvent effects (*vide infra*) may also come into play.

As outlined in Stage III of the Methods section, the application of the sTDA method to this work was validated by taking a random set of compounds with first oscillator strengths that exceed 0.8, as predicted by sTDA, and re-computing them via TD-DFT. Figure 3e shows two scatter plots displaying the correlation between the TD-DFT and sTDA computational methods for computed electronic properties, ${\lambda }_{max}^{1st}$ and *f*, using a subset of 200 compounds. Figure 3e (left) shows the comparison between two methods for ${\lambda }_{max}^{1st}$; Fig. 3e (right) shows the cognate comparison for *f*. The coefficient of determination (*R*^2^) shows very high agreement between the two methods for the computed wavelengths and good agreement for the first oscillator strength.

As stated earlier, *f* was calculated to represent the closest possible manifestation of *ϵ*, which cannot be calculated *per se*; it nonetheless relates to *ϵ* in that it presents a delta function of the absorption cross-section at a given wavelength; it does not take into account explicit solvent effects or molecular interactions, given that is arises from a gas-phase calculation. In principle, *f* can be used to calculate *ϵ* via the empirical equation: *ϵ*_*calc*_ = (*f* × 2.699 × 10^4^)/*b* where *b* is the line width of the absorption peak associated with *f* and *ϵ* ^28^. Applying this formula to the compounds in our dataset^6^ whose measurement of *ϵ* took place in the example solvent, ethanol, using *f* values from the sTDA results, affords a positive correlation with a Spearman coefficient of 0.55. Pending optimization of the line width, *b*, and the application of this relation to compounds across many types of solvent, the strength of this correlation could be improved substantially. Such optimization and wider application of this empirical relationship between *f* and *ϵ* is the subject of future work. However, the salient conclusion for this work is that a distinct correlation between *f* and *ϵ* is present, thus justifying the linkages between these two parameters in the methodology that underpins the make-up of our comparative dataset^6^.

The nature of the solvent used in UV/vis absorption spectroscopy measurements can alter *λ*_*max*_ and *ϵ* values of a compound, sometimes quite substantially owing to solvatochromic effects^29^. *λ*_*max*_ values are particularly susceptible to the extent by which the solvent involved is non-polar or polar; this scale of polarity is generally represented by the dielectric constant of the solvent which rises with increasing solvent polarity. Given the potential influence of solvent on *λ*_*max*_ and *ϵ*, the solvent used in the experimental measurement of *λ*_*max*_ for each compound in our dataset^6^ was also extracted from the scientific literature using ChemDataExtractor. Figure 3f shows a bar plot displaying the frequency of all solvents occurring at least 100 times in the dataset^6^. Only one instance of solvent is counted for each valid compound to avoid corrupting the data with multiple counts. Instances of multiplicate solvent names (e.g. Ethanol, ethanol, or EtOH) reported in the scientific literature had to be re-parsed, verified, and standardized to produce the correct count for this plot. Solvent information is presented in this plot as a function of increasing value of dielectric constant going from left to right, with the exact experimentally-determined dielectric constant being written on top of each bar. As expected for a globally representative distribution, 10 common solvents feature in the plot: toluene, chloroform, tetrahydrofuran (THF), dichloromethane (DCM), acetone, ethanol, methanol, acetonitrile, dimethylfuran (DMF) and dimethylsulfoxide (DMSO).

**Fig. 4:**
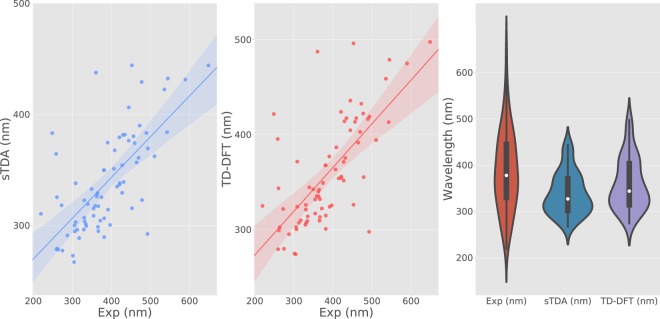
Calculated first excited-state wavelengths, ${\lambda }_{max}^{1st}$, versus *λ*_*max*_ experimental values extracted from the literature for the 76 compounds where both sTDA and TD-DFT calculations were undertaken, and only one experimental *λ*_*max*_ value was obtained. Calculations were performed using sTDA (left panel) or TD-DFT (center panel). Solid lines show the linear regression fit to each dataset and the shaded color regions show the corresponding 98% confidence interval. Mean absolute errors (MAEs) between calculated and experimental values are 64.50 nm and 51.57 nm for sTDA and TD-DFT calculations, respectively. (Right panel) Violin plot showing the wavelength distributions of experimental, sTDA and TD-DFT calculated data. White dots indicate a median; boxes show interquartile ranges; upper and lower whiskers show extremes. Any data beyond whiskers are outliers.

A sample subset of 76 entries containing sTDA, TD-DFT and experimental *λ*_*max*_ data were compared with relevant plots shown in Fig. 4. Linear trends are evident between experimental and computed wavelengths, and the distribution and scattering profile is very similar between sTDA and TD-DFT comparatives with experimental data. The data fit well within the 98% confidence interval. Stokes shifts and solvatochromic effects could easily account for the 50–65 nm differences observed between gas-phase calculations and the solution-based experimental values in this comparison i.e. the MAE values^29,30^. Thus, the data seem well within a reasonable range of comparison, given their bespoke differences (biases). The violin plots are also consistent with expectations as the wavelength distribution of *λ*_*max*_ values is notably wider than those of the computed values; the latter are delta functions and so their range is expected to be much tighter. The distributions for TD-DFT and sTDA are similar except that the TD-DFT data portray a tail in the redder region of wavelengths. The statistics associated with the violin plot are displayed in Table 2.

**Table 2:** Distribution statistics of data associated with Fig. 4.

Metric	Exp (nm)	sTDA (nm)	TD-DFT (nm)
Median	378	327	344
Upper quartile	445	371	403
Lower quartile	331	303	315

The medians of the simulated data are 17 nm apart, with the TD-DFT distribution median (344 nm) closer to the experimental median (378 nm). The relative shift between sTDA and TD-DFT at the lower quartile and upper quartile are 12 nm and 32 nm, respectively. Qualitatively, it can observed that TD-DFT slightly improves in higher wavelength (i.e. upper quartile) regions relative to sTDA. The difference in the upper quartile for TD-DFT can be inferred as the reason for the decrease in MAE by 13 nm.

Overall, the results of our technical validation indicate that our auto-generated dataset of UV/vis absorption spectral attributes^6^ is representative of the wavelength distribution for organic compounds. Moreover, we have shown that the cognate *λ*_*max*_ and *f* values calculated via our use of the fast computational method, sTDA, are deemed to be reliable. *ϵ* and *f* seem to be comparable metrics that relate experiment and computation. Representative solvent information is captured. Our dataset^6^ affords the largest, openly available source of UV/vis absorption spectral attributes, *λ*_*max*_ and *ϵ*, to date. It also presents a rare example of a dataset that contains paired quantities of cognate experimental and computational physical properties. Amongst other things, the availability of these matching experimental and computational data lays the path for reliable *in silico* calculations of additional optical properties as well as other properties.

## Usage Notes

The datasets^6^ are available in MongoDB, JSON and CSV formats. The most relevant information pertinent to the dataset are provided in the CSV format. Due to the unstructured nature of our dataset^6^, the expanded information, which includes detailed parsed calculation outputs from each stage, had to be stored in ‘non relational’ style JSON data format and in a MongoDB management framework. These can be queried using Python, R, Java, and MATLAB, for data-driven optoelectronic materials discovery. These programming options were selected since they cover the most popular range of scripting, statistical, web-based and scientific computing platforms in use by physical scientists. Additionally, a good variety of wrappers exist for converting these capabilities between programming platforms. Instructions on using the MongoDB query language can be found online at https://docs.mongodb.com/v3.4/core/document/.

## Supplementary information

Supplementary Information

## Data Availability

The compound data were extracted from the scientific literature using a UV/vis absorption spectroscopy tailored version of ChemDataExtractor, which is available at https://github.com/edbeard/chemdataextractor-uvvis2018. A clean build of the current release of ChemDataExtractor version 1.3 can be found at http://chemdataextractor.org/download. The scripts used to filter the data to leave chemically valid compounds are available alongside the database^6^ at 10.6084/m9.figshare.7619672.v2 in the ‘scripts.zip’ directory. Scripts used for the QMWF pipeline in Stage II can be found at https://github.com/alvarovm/qmwf.
