# Supplementary material for: Comparative dataset of experimental and computational attributes of UV/vis absorption spectra
Source: Sci Data. 2019 Dec 5;6:307. doi: 10.1038/s41597-019-0306-0 (PMC6895184; doi:10.1038/s41597-019-0306-0)
Supplement: Supplementary file 1 — Supplementary Information [file 41597_2019_306_MOESM1_ESM.pdf]

Supplementary Information

Below is a detailed list of the scientific journals chosen for extraction alongside their publisher. Journals were selected by an expert in the field.

Bioorganic Medicinal Chemistry Letters - *Elsevier*

Bioorganic Medicinal Chemistry - *Elsevier*

Carbohydrate Research - *Elsevier*

Catalysis Today - *Springer*

Chemical Communications - *RSC*

Chemical Data Collections - *Elsevier*

Chemical Physics - *Elsevier*

Chemical Science - *RSC*

Chinese Chemical Letters - *Elsevier*

Comptes Rendus Chimie - *Elsevier*

Dyes And Pigments - *Elsevier*

Electrochemistry Communications - *Elsevier*

Inorganic Chemistry Communications - *Elsevier*

Inorganica Chimica Acta - *Elsevier*

Journal of Electroanalytical Chemistry - *Elsevier*

Journal of Fluorine Chemistry - *Elsevier*

Journal of Luminescence - *Elsevier*

Journal of Materials Chemistry A - *RSC*

Journal of Materials Chemistry B - *RSC*

Journal of Materials Chemistry C - *RSC*

Journal of Molecular Liquids - *Elsevier*

Journal of Molecular Structure - *Elsevier*

Journal of Organometallic Chemistry - *Elsevier*

Journal of Photochemistry and Photobiology A : Chemistry - *Elsevier*

Journal of Solid State Chemistry - *Elsevier*

Materials Characterization - *Elsevier*

Microchemical Journal - *Elsevier*

Optical Materials - *Elsevier*

Physical Chemistry Chemical Physics - *RSC*

Polyhedron - *Elsevier*

Solid State Sciences - *Elsevier*

Tetrahedron: Asymmetry - *Elsevier*

Tetrahedron - *Elsevier*
